# Prevalence and associated factors of incident and persistent loneliness among middle-aged and older adults in Thailand

**DOI:** 10.1186/s40359-023-01115-4

**Published:** 2023-03-14

**Authors:** Supa Pengpid, Karl Peltzer

**Affiliations:** 1 Department of Health Education and Behavioral Sciences, Faculty of Public Health, Mahidol University, Bangkok, Thailand; 2 Department of Public Health, Sefako Makgatho Health Sciences University, Pretoria, South Africa; 3 Department of Healthcare Administration, College of Medical and Health Science, Asia University, Taichung, Taiwan; 4 Department of Psychology, University of the Free State, PO Box 339 (40), 9300 Bloemfontein, South Africa; 5 Department of Psychology, College of Medical and Health Science, Asia University, Taichung, Taiwan

**Keywords:** Lifestyle factors, Chronic diseases, Loneliness, Prospective cohort study, Thailand

## Abstract

**Background:**

The aim of the study was to assess the prevalence and associated factors of incident and persistent loneliness in a prospective cohort study among middle-aged and older adults (≥ 45 years) in Thailand.

**Methods:**

Longitudinal data from the Health, Aging, and Retirement in Thailand (HART) study in 2015 and 2017 were analysed. Loneliness was assessed with one item from the Center for Epidemiological Studies Depression scale. Logistic regression was used to calculate predictors of incident and persistent loneliness.

**Results:**

In total, at baseline 21.7% had loneliness, 633 of 3696 participants without loneliness in 2015 had incident loneliness in 2017 (22.2%), and 239 of 790 adults had persistent loneliness (in both 2015 and 2017) (30.3%). In adjusted logistic regression analysis, low income (aOR: 1.27, 95% CI: 1.03 to 1.57), poor self-rated physical health status (aOR: 1.64, 95% CI: 1.27 to 2.12), hypertension (aOR: 1.34, 95% CI: 1.09 to 1.65), depressive symptoms (aOR: 1.97, 95% CI: 1.11 to 3.49), and having three or chronic conditions (aOR: 1.76, 95% CI: 1.19 to 2.60) were positively associated and a higher education (aOR: 0.74, 95% CI: 0.55 to 0.98) and living in the southern region of Thailand (aOR: 0.43, 95% CI: 0.30 to 0.61) were inversely associated with incident loneliness. Poor self-rated physical health status (aOR: 1.91, 95% CI: 1.26 to 2.88), and having three or more chronic diseases (aOR: 1.78, 95% CI: 1.07 to 2.98), were positively associated, and living in the southern region (aOR: 0.40, 95% CI: 0.25 to 0.65) was inversely associated with persistent loneliness.

**Conclusion:**

More than one in five ageing adults had incident loneliness in 2 years of follow-up. The prevalence of incident and/or persistent loneliness was higher in people with a lower socioeconomic status, residing in the central region, poor self-rated physical health status, depressive symptoms, hypertension, and a higher number of chronic diseases.

## Introduction

Loneliness may interfere with the quantity and quality of one’s social relationships [1] and may be common among older adults as their social relations may decline [2, 3]. Loneliness affects negatively physical and mental health in old age, including mortality [4–6]. The prevalence of loneliness among older adults in high-income countries was 28.5% [7]. Among middle-aged and older adults in low- and middle-income countries, the prevalence of loneliness was 33.8% in India [8], in South Africa 9.9% [9], 10% in Indonesia [10], in Malaysia 32.5% [11], in Mexico 12.3% [12], and in Myanmar (31.7%) [14]. In local studies in Thailand in the Ko Rai Subdistrict, Chachoengsao, 24% of older adults reported loneliness [13], among older Adults in Chon Buri Province, a low level of loneliness was found [15], and in a qualitative study in Thailand contributing factors to loneliness included life transitions, socio-demographic factors, socio-economic and environmental factors, poor health status and health resources [16]. The prevalence of incident loneliness (4 years) among middle-aged and older adults in Indonesia was 15.1% [17], and in the English Longitudinal Study of Ageing (ELSA) 38.4% (length of follow-up 6.86 years), and the Health and Retirement Study (HRS) in USA 30.2% (length of follow-up 8.18 years) [18].

Considering that previous local studies on loneliness in Thailand were cross-sectional, the prevalence of incident and persistent loneliness symptoms among the ageing population in Thailand is unclear, as well as the prospective relationships between baseline indicators and incident and persistent loneliness. A greater understanding of the prevalence and associated factors of incident and persistent loneliness may help in better identifying and treating modifiable risk factors in people with loneliness.

In a systematic review of longitudinal studies in high-income countries among older adults found the following risk factors for loneliness, not married, low social activity, poor self-rated health; and depression [19]. In a systematic review of mainly cross-sectional studies among older adults, female sex, older age, lower socioeconomic status, non-married status, living alone, functional disability, poor self-report physical and mental health, poor cognitive functioning, and adverse life events were associated with loneliness [20].

There is a lack of longitudinal studies in Southeast Asia investigating the determinants of incident and persistent loneliness. To address this research gap, our objective was to investigate the prevalence and associated factors of incident and persistent loneliness in a prospective cohort study among ageing adults (≥ 45 years) in Thailand.

## Methods

### Sample and procedure

Secondary longitudinal data from the Health, Aging, and Retirement in Thailand (HART) study in 2015 and 2017 were analysed. In a national multi-stage sampling (regions, provinces, blocks or villages, households) one individual (≥ 45 years) was randomly selected per household [21, 22]. The 2015 study (N = 5,616), and the 2017 study (N = 3,708) had a 72.3% response rate and 66.0% the retention rate; reasons for loss to follow-up were had moved away (N = 1,554), declined (N = 270) and died (N = 192).

Participants were interviewed with a structured questionnaire in 2015 and with computer-assisted personal interview (CAPI) in 2017. The “Ethics Committee in Human Research, National Institute of Development Administration – ECNIDA (ECNIDA 2020/00012)” granted approval, and participants gave written informed consent.

## Measures

### Outcome variable

*Loneliness* was measured with one item from the “Center for Epidemiologic Studies Depression (CES-D-10) scale,” [23], “In the past week, how often did you experience feeling lonely?” defined as “almost always (5–7 days), often (3–4 days) or sometimes (1–2 days)”=1 and “very rarely (less than one day) or none”=0. Single-item measures of loneliness have shown adequate reliability and have a high correlation with multi-item measures [24–26].

### Covariates

*Sociodemographic variables* included marital status, highest level of education (no formal education illiterate/ no formal education literate, or elementary school, coded as “≤elementary school”, and middle school, high school, vocational diploma, Bachelor degree, or higher than Bachelor degree, coded as “>elementary school”), sex, age, region, religion, and personal annual income. The personal annual income was calculated based from “employment, own business, agricultural/livestock/fishing business, short-term or contract work, financial support from family, remuneration/pension income from the government fund, occupational pension fund, private pension fund, social security/welfare income, income from government living allowance, veteran’s welfare benefit, other welfare assistance income, and income from other sources, and classified low = less than 50,000 Baht, and high = 50,000 or more Thai Baht (Average exchange rate in 2015: 1 US = 34.2 Baht)” [22].

*Social participation* (at least one social activity in the past month) was sourced from 6 items [22, 27].

*Substance use* included alcohol use and smoking (never, past, or current). “Have you ever smoked cigarettes?” (response options: “1 = yes, and still smoke now, 2 = yes, but quit smoking, and 3 = never”); “Have you ever drunk alcoholic beverages such as liquor, beer, or wine?” (response options: “1 = yes, and still drinking now, 2 = yes, but do not drink now, and 3 = never)”.

*Physical activity* was classified as 0–149 min/week exercise, and ≥ 150 min/week exercise [28, 29].

*Body Mass Index* (BMI), based on self-reported height and weight: “underweight (< 18.5 kg/m^2^), normal weight (18.5–22.9 kg/m^2^), overweight (23–24.9 kg/m^2^), and obesity (25 + kg/m^2^).” [30].

*Activities of daily living (ADL) disability* was defined as the inability to do any of the four ADL (eating, bathing, dressing, and washing) [31].

*History of accidents* (injury) was assessed with the question, “In the last 2 years, were you involved in an accident that affected your physical health?” (Yes/No).

*Fear of falling (FOF) with activity avoidance* was defined as “so worried about falling down that I have refrained from doing certain activities.”

*The self-rated physical health status* (ranging from 0 = very poor to 100 excellent) was defined as 0–50 low and 60–100 high.

*Depressive symptoms* (≥ 10 scores) was measured with the “Center for Epidemiologic Studies Depression (CES-D-10) scale,” [23], excluding the loneliness item [32, 33]; (Cronbach’s alpha 0.76).

*Chronic diseases* were based on self-reported conditions that had been diagnosed by a health care professional, including: “1) hypertension, 2) diabetes, 3) vascular diseases, heart disease or heart failure, 4) rheumatism or arthritis, 5) bone diseases, low bone density or osteoporosis, 6) kidney diseases, 7) lung diseases/emphysema, 8) cancer, 9) liver diseases, 10) brain diseases/Alzheimer’s disease 11) visual impairment and 12) hearing impairment.” The 12 chronic diseases were classified into nine groups: (1) cardiovascular disease, heart disease, heart failure, (2) hypertension, (3) endocrine (diabetes), (4) musculoskeletal (bone diseases, rheumatism, low bone density, arthritis, and osteoporosis), (5) liver or kidney diseases, (6) respiratory (lung diseases/emphysema), (7) Cancer, (8) sensorial (visual impairment and/or hearing impairment), and (9) neurological (brain diseases/Alzheimer’s disease) [34].

### Statistical analysis

Frequencies and percentages of incident and persistent loneliness were calculated. The first longitudinal logistic regression model estimated incident loneliness in 2017, excluding those with loneliness in 2015, and the second model estimated persistent loneliness (in both 2015 and 2017). Models were adjusted by chronic diseases, sociodemographic factors, lifestyle factors, social participation, depressive symptoms, and adverse life events; confounders were included based on literature review [19, 20]. Variables found significant at < 0.05 in univariate analyses were included in the multivariable models; p < 0.05 was considered statistically significant. Missing data were discarded. Statistical analyses were conducted with StataSE 15.0 (College Station, TX, USA).

## Results

### Sample characteristics

In total, at baseline 21.7% had loneliness, 633 of 3696 participants without loneliness in 2015 had incident loneliness in 2017 (22.2%), and 239 of 790 adults had persistent loneliness (in both 2015 and 2017) (30.3%). In addition, the details of the sample are shown in Table 1.

**Table 1:** Analytic sample characteristics by incident and persistent loneliness, Thailand, 2015–2017

Baseline variables	Subcategories	Baseline sample	Incident loneliness	Persistent loneliness
Total N		3696	2855	790
		N (%)	N (%)	N (%)
All			633 (22.2)	239 (30.3)
Age (in years)	45–64 ≥ 65 or more	1685 (45.6) 2011 (54.4)	246 (18.1) 387 (25.9)	90 (29.8) 149 (30.5)
Sex	Female Male	1971 (53.3) 1725 (46.7)	356 (23.8) 277 (20.3)	154 (34.3) 85 (24.9)
Education	≤Elementary >Elementary	3100 (84.1) 588 (15.9)	555 (23.4) 75 (15.8)	213 (31.2) 26 (24.5)
Annual income	High Low	1875 (50.7) 1821 (49.3)	286 (18.4) 347 (26.6)	86 (28.0) 153 (31.7)
Marital status	Married/cohabiting Divorced/sep./never married Widowed	2194 (60.1) 352 (9.6) 1105 (30.3)	352 (19.8) 56 (22.3) 213 (26.8)	105 (27.2) 27 (27.8) 103 (34.7)
Religion	Muslim or other Buddhist	304 (8.2) 3390 (91.8)	36 (19.4) 597 (22.4)	28 (24.8) 211 (31.2)
Region	Bangkok Central (excl. Bangkok) North Northeast South	287 (7.8) 899 (24.3) 1126 (30.5) 659 (17.8) 725 (19.6)	66 (28.7) 170 (23.1) 207 (23.4) 128 (23.1) 62 (13.7)	21 (38.2) 66 (42.0) 71 (31.8) 33 (35.1) 48 (18.4)
Social participation	No Yes	241 (6.5) 3450 (93.5)	46 (24.0) 587 (22.0)	16 (34.0) 223 (30.1)
Alcohol use	Never Past Current	2988 (80.8) 266 (7.2) 442 (12.0)	523 (22.8) 47 (23.6) 63 (17.5)	205 (31.7) 17 (25.8) 17 (22.1)
Smoking tobacco use	Never Past Current	293 (79.9) 292 (7.9) 451 (12.2)	515 (22.7) 45 (20.2) 73 (20.2)	199 (31.1) 19 (28.8) 21 (25.0)
Physical activity	≥ 150 min/week < 150 min/week	605 (16.4) 3091 (83.6)	100 (20.2) 533 (22.6)	38 (36.5) 201 (29.3)
Body mass index	Normal Under Overweight Obesity	1247 (37.6) 354 (10.7) 661 (19.9) 1057 (31.8)	226 (23.1) 70 (27.1) 112 (20.6) 171 (20.6)	81 (31.9) 29 (31.5) 23 (21.1) 73 (33.2)
Activities of Daily Living disability	No Yes	3522 (96.9) 112 (3.1)	606 (21.8) 18 (37.5)	218 (30.4) 19 (29.7)
Accident	No Yes	3249 (87.9) 447 (12.1)	544 (21.6) 89 (26.4)	201 (29.5) 38 (35.2)
Fall worry refrain from social activities	No Yes	3425 (92.7) 271 (7.3)	587 (21.7) 46 (30.7)	203 (30.3) 36 (29.8)
Self-rated physical health status	High Low	3024 (83.4) 600 (16.6)	489 (20.3) 137 (33.8)	162 (27.7) 74 (40.7)
Depressive symptoms	No Yes	3118 (91.6) 285 (8.4)	576 (22.0) 22 (40.0)	147 (29.6) 71 (31.0)
**Chronic conditions**				
Cardiovascular disease	No Yes	3499 (94.7) 197 (5.3)	590 (21.8) 43 (29.1)	219 (29.5) 20 (42.6)
Hypertension	No Yes	2379 (64.4) 1317 (35.6)	358 (19.1) 275 (28.0)	136 (29.2) 103 (31.8)
Endocrine (diabetes)	No Yes	3139 (84.9) 557 (15.1)	527 (21.6) 106 (25.5)	192 (29.4) 47 (34.6)
Musculoskeletal	No Yes	3434 (92.9) 262 (7.1)	573 (21.4) 60 (33.1)	211 (29.6) 28 (35.9)
Liver or kidney diseases	No Yes	3608 (97.6) 88 (2.4)	620 (22.2) 13 (21.7)	232 (30.4) 7 (25.9)
Respiratory	No Yes	3660 (99.0) 36 (1.0)	627 (22.2) 6 (22.2)	236 (30.2) 3 (33.3)
Cancer	No Yes	3664 (99.1) 32 (0.9)	626 (22.1) 7 (31.8)	235 (30.1) 4 (40.0)
Sensorial	No Yes	3129 (84.7) 567 (15.3)	530 (21.5) 103 (26.5)	189 (28.9) 59 (35.3)
Neurological	No Yes	3665 (99.2) 31 (0.8)	626 (22.1) 7 (43.8)	234 (30.2) 5 (35.7)
**Number of chronic conditions**				
Chronic conditions	0 1 2 3 or more	1716 (46.4) 1101 (29.8) 600 (16.2) 279 (7.5)	246 (17.8) 205 (24.3) 122 (27.5) 60 (33.0)	80 (26.4) 76 (31.3) 45 (30.2) 38 (40.0)

Sep.= separated

### Associations with incident loneliness

In adjusted logistic regression analysis, low income (aOR: 1.27, 95% CI: 1.03 to 1.57), poor self-rated physical health status (aOR: 1.64, 95% CI: 1.27 to 2.12), depressive symptoms (aOR: 1.97, 95% CI: 1.11 to 3.49), hypertension (aOR: 1.34, 95% CI: 1.09 to 1.65) and having three or chronic conditions (aOR: 1.76, 95% CI: 1.19 to 2.60) were positively associated and a higher education (aOR: 0.74, 95% CI: 0.55 to 0.98) and living in the southern region of Thailand (aOR: 0.43, 95% CI: 0.30 to 0.61) were inversely associated with incident loneliness. Furthermore, in the unadjusted analysis, older age, widowed, ADL disability, having had an accident in the past two years, fear of falling with refraining from social activities, cardiovascular disease, musculoskeletal problem, sensory and neurological conditions were positively associated with incident loneliness, while male sex, social participation, and current alcohol use were negatively associated with incident loneliness (see Table 2).

**Table 2:** Association between independent variables and incident loneliness, HART (2015–2017)

Baseline variables	Subcategory	COR (95% CI)	AOR (95% CI)
Age (in years)	45–64 ≥ 65 or more	1 (Reference) 1.58 (1.32 to 1.89)***	1 (Reference) 1.16 (0.93 to 1.45)
Sex	Female Male	1 (Reference) 0.82 (0.68 to 0.97)*	1 (Reference) 0.98 (0.79 to 1.23)
Education	≤Elementary >Elementary	1 (Reference) 0.62 (0.47 to 0.80)***	1 (Reference) 0.74 (0.55 to 0.98)*
Annual income	High Low	1 (Reference) 1.61 (1.35 to 1.92)***	1 (Reference) 1.27 (1.03 to 1.57)*
Marital status	Married/cohabiting Divorced/sep./never married Widowed	1 (Reference) 1.16 (0.84 to 1.60) 1.48 (1.21 to 1.80)***	1 (Reference) 1.07 (0.75 to 1.51) 1.13 (0.89 to 1.43)
Religion	Muslim or other Buddhist	1 (Reference) 1.20 (0.83 to 1.75)	---
Region	Central North Northeast South	1 (Reference) 0.95 (0.76 to 1.17) 0.93 (0.72 to 1.19 0.49 (0.36 to 0.66)***	1 (Reference) 0.88 (0.70 to 1.12) 0.97 (0.74 to 1.27) 0.43 (0.30 to 0.61)***
Social participation	No Yes	1 (Reference) 0.74 (0.62 to 0.90)**	1 (Reference) 0.96 (0.67 to 1.40)
Alcohol use	Never Past Current	1 (Reference) 1.05 (0.75 to 1.48) 0.72 (0.54 to 0.96)*	1 (Reference) 0.95 (0.64 to 1.41) 0.86 (0.62 to 1.20)
Smoking tobacco use	Never Past Current	1 (Reference) 0.86 (0.61 to 1.21) 0.86 (0.66 to 1.14)	---
Physical activity	≥ 150 min/week < 150 min/week	1 (Reference) 1.16 (0.91 to 1.47)	---
Body mass index	Normal Under Overweight Obesity	1 (Reference) 1.24 (0.91 to 1.70) 0.86 (0.67 to 1.11) 0.87 (0.69 to 1.09)	---
Activities of Daily Living disability	No Yes	1 (Reference) 2.15 (1.19 to 3.88)*	1 (Reference) 1.51 (0.78 to 2.91)
Accident	No Yes	1 (Reference) 1.60 (1.17 to 2.19)**	1 (Reference) 1.22 (0.92 to 1.63)
Fall worry refrain from social activities	No Yes	1 (Reference) 1.60 (1.12 to 2.29)*	1 (Reference) 1.12 (0.75 to 1.87)
Self-rated physical health status	High Low	1 (Reference) 2.01 (1.60 to 2.52)***	1 (Reference) 1.64 (1.27 to 2.12)***
Depressive symptoms	No Yes	1 (Reference) 2.36 (1.36 to 4.08)**	1 (Reference) 1.97 (1.11 to 3.49)*
**Chronic conditions**			
Cardiovascular disease	No Yes	1 (Reference) 1.47 (1.02 to 2.12)*	1 (Reference) 0.99 (0.65 to 1.50)
Hypertension	No Yes	1 (Reference) 1.65 (1.37 to 1.97)***	1 (Reference) 1.34 (1.09 to 1.65)**
Endocrine (diabetes)	No Yes	1 (Reference) 1.24 (0.98 to 1.58)	---
Musculoskeletal	No Yes	1 (Reference) 1.82 (1.32 to 2.51)***	1 (Reference) 1.61 (1.12 to 2.30)
Liver or kidney diseases	No Yes	1 (Reference) 0.97 (0.52 to 1.81)	---
Respiratory	No Yes	1 (Reference) 1.00 (0.40 to 2.45)	---
Cancer	No Yes	1 (Reference) 1.65 (0.67 to 4.05)	---
Sensorial	No Yes	1 (Reference) 1.32 (1.03 to 1.68)*	1 (Reference) 1.05 (0.79 to 1.40)
Neurological	No Yes	1 (Reference) 2.75 (1.02 to 7.41)*	1 (Reference) 1.60 (0.48 to 5.39)
**Number of chronic conditions**			
Chronic conditions	0 1 2 3 or more	1 (Reference) 1.48 (1.20 to 1.83)*** 1.76 (1.37 to 2.26)*** 2.28 (1.62 to 3.19)***	1 (Reference)^a^ 1.28 (1.02 to 1.61)* 1.40 (1.06 to 1.85)* 1.76 (1.19 to 2.60)**

Sep.= separated; COR = Crude Odds Ratio; AOR = Adjusted Odds Ratio; ***p < 0.001;**p < 0.01; *p < 0.05; ^a^adjusted for all variables except for individual chronic conditions

### Associations with persistent loneliness

In adjusted logistic regression analysis, poor self-rated physical health status (aOR: 1.91, 95% CI: 1.26 to 2.88), and having three or more chronic conditions (aOR: 1.78, 95% CI: 1.07 to 2.98), were positively associated and living in the southern region (aOR: 0.40, 95% CI: 0.25 to 0.65) was negatively associated with persistent loneliness. In addition, in univariable analysis, widowed was positively associated and male sex, and living in the northern region were negatively associated with persistent loneliness (see Table 3).

**Table 3:** Associations between independent variables and persistent loneliness, HART (2015–2017)

Baseline variables	Subcategory	COR (95% CI)	AOR (95% CI)
Age (in years)	45–64 ≥ 65 or more	1 (Reference) 1.04 (0.76 to 1.42)	---
Sex	Female Male	1 (Reference) 0.64 (0.47 to 0.87)**	1 (Reference) 0.71 (0.49 to 1.03)
Education	≤Elementary >Elementary	1 (Reference) 0.72 (0.45 to 1.14)	---
Annual income	High Low	1 (Reference) 1.19 (0.87 to 1.63)	---
Marital status	Married/cohabiting Divorced/sep./never married Widowed	1 (Reference) 1.03 (0.63 to 1.70) 1.42 (1.02 to 1.97)*	1 (Reference) 0.78 (0.44 to 1.38) 1.16 (0.79 to 1.70)
Religion	Muslim or other Buddhist	1 (Reference) 1.38 (0.87 to 2.18)	---
Region	Central North Northeast South	1 (Reference) 0.67 (0.45 to 0.99)* 0.77 (0.47 to 1.28) 0.32 (0.21 to 0.49)***	1 (Reference) 0.74 (0.47 to 1.14) 0.87 (0.49 to 1.54) 0.40 (0.25 to 0.65)***
Social participation	No Yes	1 (Reference) 0.83 (0.45 to 1.56)	---
Alcohol use	Never Past Current	1 (Reference) 0.75 (0.42 to 1.33) 0.61 (0.35 to 1.07)	---
Smoking tobacco use	Never Past Current	1 (Reference) 0.90 (0.51 to 1.57) 0.74 (0.44 to 1.24)	---
Physical activity	≥ 150 min/week < 150 min/week	1 (Reference) 0.72 (0.47 to 1.11)	---
Body mass index	Normal Under Overweight Obesity	1 (Reference) 0.98 (0.59 to 1.64) 0.57 (0.34 to 0.97)* 1.06 (0.72 to 1.56)	1 (Reference) 0.85 (0.49 to 1.46) 0.58 (0.33 to 1.01) 0.95 (0.63 to 1.44)
Activities of Daily Living disability	No Yes	1 (Reference) 0.97 (0.55 to 1.69)	---
Accident	No Yes	1 (Reference) 1.30 (0.85 to 1.99)	---
Fall worry refrain from social activities	No Yes	1 (Reference) 0.97 (0.64 to 1.48)	---
Self-rated physical health status	High Low	1 (Reference) 1.79 (1.27 to 2.53)***	1 (Reference) 1.91 (1.26 to 2.88)**
Depressive symptoms	No Yes	1 (Reference) 1.07 (0.76 to 1.50)	---
**Chronic conditions**			
Cardiovascular disease	No Yes	1 (Reference) 1.77 (0.97 to 2.23)	---
Hypertension	No Yes	1 (Reference) 1.13 (0.83 to 1.54)	---
Endocrine (diabetes)	No Yes	1 (Reference) 1.27 (0.86 to 1.88)	---
Musculoskeletal	No Yes	1 (Reference) 1.33 (0.82 to 2.17)	---
Liver or kidney diseases	No Yes	1 (Reference) 0.80 (0.33 to 1.92)	---
Respiratory	No Yes	1 (Reference) 1.16 (0.29 to 4.66)	---
Cancer	No Yes	1 (Reference) 1.55 (0.43 to 3.88)	---
Sensorial	No Yes	1 (Reference) 1.34 (0.94 to 1.93)	---
Neurological	No Yes	1 (Reference) 1.29 (0.43 to 3.88)	---
**Number of chronic conditions**			
Chronic conditions	0 1 2 3 or more	1 (Reference) 1.27 (0.87 to 1.84) 1.21 (0.78 to 1.86) 1.86 (1.15 to 3.01)*	1 (Reference)^a^ 1.23 (0.83 to 1.82) 1.21 (0.76 to 1.91) 1.78 (1.07 to 2.98)*

Sep.=separated; COR = Crude Odds Ratio; AOR = Adjusted Odds Ratio; ***p < 0.001;**p < 0.01; *p < 0.05; ^a^adjusted for all variables except for individual chronic conditions

## Discussion

In this first prospective cohort study in among ageing adults in Thailand, we found that the prevalence of incident loneliness in a 2-year follow-up was 22.2%, which is higher than among ageing adults (4-year follow-up) in Indonesia (15.1%) [17], and lower than in ELSA (38.4%) (length of follow-up 6.86 years), and the HRS (30.2%) (length of follow-up 8.18 years) [18]. The cross-sectional prevalence of loneliness was 21.7% in this study, which is lower than in a community study in persons aged 60 years and older in the Ko Rai subdistrict, Chachoengsao, Thailand (24%) [13], in Myanmar (31.7%) [14], Malaysia (32.5%) [11], in India (33.8%) [8] but lower than in Mexico (12.3%) [12] and South Africa (9.9%) [9]. This study showed that loneliness is a significant public health issue in Thailand, calling for intervention programmes to reduce the burden of loneliness.

We found that lower economic status, lower education, living in the central region, poor self-rated physical health, depressive symptoms, hypertension, and a higher number of chronic diseases were associated with incident loneliness. In addition, living in the central region, poor self-rated physical health, and having three or more chronic conditions were associated with persistent loneliness. The observed associations were in univariate analysis higher among females, widowed, increased age and those who had no social participation.

Consistent with previous longitudinal and cross-sectional reviews [19, 20], we found that lower socioeconomic status, depressive symptoms, and poor self-perceived health were associated with loneliness. People with lower socioeconomic status may have less access to social engagement and activities, which may increase loneliness [10, 20, 35]. Depressed mood and poor self-rated health status may be closely linked with loneliness and its relationship is complex and probably bidirectional [36–38]. In addition, in agreement with previous reviews [19, in high-income countries from 1999 to 2018; 20, except for one study in Nepal, all other studies from high income countries, from 2000 to 2012], in univariate analysis, female sex, widowed, no social participation, ADL disability, adverse life event (accident, fear of falling with activity avoidance) were associated with incident loneliness. We found a lower risk of loneliness among older adults living in the southern region of Thailand. However, when calculating the baseline loneliness prevalence, we found a significantly higher prevalence of loneliness in the southern region (36.5%) than in the central region (18.0%). Meaning that the southern region had a higher baseline prevalence of loneliness than in the other regions, but the southern region had a lower prevalence of incident and persistent loneliness than in the other regions. The high baseline prevalence of loneliness in the southern region may be explained by lower economic indicators [22], lower health care utilization in older rural than urban dwellers [39], and compared to Bangkok older adults in rural areas have a disadvantage in healthcare due to lower socioeconomic capacity and lower healthcare access [40]. On the other hand, the lower prevalence of incident and persistent loneliness in the southern region of Thailand, may be attributed to the southern region having the highest number of Muslims and may be more rural than other regions, while the highest rate of loneliness was in the central region, which is the most urbanized region, all of which are factors that increase loneliness [41]. A previous study found a negative correlation between perceived social support and loneliness among older Muslims [42].

Furthermore, the existence of multiple chronic diseases was found to be associated with incident and persistent loneliness. In a longitudinal study in Germany, the multimorbidity was associated with incident loneliness [43], and in a cross-sectional study in the UK, physical multimorbidity was in a dose response fashion associated with loneliness [44]. The development of loneliness may be explained by limiting participation in activities and dependency feelings due to multimorbidity [9, 10, 45, 46]. In particular, hypertension was found to be associated with incident loneliness in this study. Previous studies showed that loneliness was a risk factor for hypertension [e.g., 47], or it could also be bidirectional [48].

In univariate analysis musculoskeletal conditions, cardiovascular disease, sensory loss and neurological problems were association with incident loneliness. Musculoskeletal conditions are often associated with body pain, and Emerson et al. [49] found in a longitudinal study that pain was a risk factor for loneliness among older adults, while in another study bidirectional longitudinal associations were found between loneliness and pain [50]. In a cross-sectional annual survey having had a stroke was associated with higher levels of loneliness [51]. However, in a longitudinal study in England there was an association between coronary heart disease and incident loneliness, but this became non-significant in the adjusted analysis [52]. Persistent loneliness increased the risk of developing dementia and Alzheimer’s disease [53]. In a longitudinal study in China, sensory impairment increased the risk of loneliness [54], and in a systematic review hearing loss increased the odds of loneliness [55]. Ageing adults with impaired vision and/or hearing may be more likely to experience ADL disability and poor social support, which can lead to incident loneliness.

Unlike some previous research (in terms of substance use [56] and physical inactivity [57], our study did not find a significant association between smoking, alcohol use, and physical inactivity with incident and persistent loneliness. Similarly to a longitudinal study in Germany [58], this survey was unable to find a significant association between obesity and loneliness.

### Study limitations

A limitation of the study was a large proportion of loss of follow-up (32%). Like in many previous studies, we evaluated loneliness with a single item, which, however, has shown high correlations with multi-item loneliness measures [24–26]. Some variables, such as living status and cognitive function, were not assessed, and should become part of future research. Furthermore, the study used a screening questionnaire for depression. The follow-up period (2 years) was relatively short, and longer repeated follow-ups may be needed to identify stronger results.

## Conclusion

More than one in five ageing adults had incident loneliness in 2 years of follow-up. The prevalence of incident and/or persistent loneliness was higher in people with a lower socioeconomic status, residing in the central region, poor self-rated physical health status, depressive symptoms, hypertension, and a higher number of chronic diseases. Results show the importance of baseline health status indicators in relation to impacting longitudinal changes in loneliness. Identifying individuals with the identified risk factors can help in providing early interventions to prevent the development of loneliness.

## Data Availability

Data is publicly available at Gateway to Global Ageing Data, Health, Aging, and Retirement in Thailand: https://g2aging.org/?section=study&studyid=44.
